# Improvement of protein production in baculovirus expression vector system by removing a total of 10 kb of nonessential fragments from *Autographa californica multiple nucleopolyhedrovirus* genome

**DOI:** 10.3389/fmicb.2023.1171500

**Published:** 2023-04-13

**Authors:** Xiaoyue Zhang, Aiping He, Yuyu Zong, Houlu Tian, Zhihui Zhang, Kaixia Zhao, Xiaodong Xu, Hongying Chen

**Affiliations:** College of Life Sciences, Northwest A&F University, Yangling, Shaanxi, China

**Keywords:** baculovirus expression vector system, *Autographa californica multiple nucleopolyhedrovirus*, non-essential genes, protein production, gene knockout

## Abstract

Baculovirus expression vector system (BEVS) is a powerful and versatile platform for recombinant protein production in insect cells. As the most frequently used baculovirus, *Autographa californica multiple nucleopolyhedrovirus* (AcMNPV) encodes 155 open reading frames (ORFs), including a considerable number of non-essential genes for the virus replication in cell culture. Studies have shown that protein production in BEVS can be improved by removing some viral dispensable genes, and these AcMNPV vectors also offer the possibility of accommodating larger exogenous gene fragments. In this study, we, respectively, deleted 14 DNA fragments from AcMNPV genome, each of them containing at least two contiguous genes that were known nonessential for viral replication in cell culture or functionally unknown. The effects of these fragment-deletions on virus replication and exogenous protein production were examined. The results showed that 11 of the 14 fragments, containing 43 genes, were dispensable for the virus replication in cultured cells. By detecting the expression of intracellularly expressed and secreted reporter proteins, we demonstrated that nine of the fragment-deletions benefited protein production in *Sf*9 cells and/or in High Five cells. After combining the deletion of some dispensable fragments, we obtained two AcMNPV vectors shortened by more than 10 kb but displayed an improved capacity for recombinant protein production. The deletion strategies used in this study has the potential to further improve the BEVS.

## 1. Introduction

Baculoviruses are a group of double-stranded DNA viruses with varied genome sizes from 80 to 180 kb. Among these viruses, the most well studied species is *Autographa californica multiple nucleopolyhedrovirus* (AcMNPV) (Rohrmann, 2019). Since the first successful utilization of AcMNPV in the expression of human β-interferon in insect cells (Smith et al., 1983), the baculovirus expression vector system (BEVS) has been used for the production of a large number of exogenous proteins. BEVS is a powerful platform for the production of functional eukaryotic proteins that require post-translational modifications such as glycosylation and phosphorylation. AcMNPV has a genome size of about 134 kb which can accommodate large foreign genes. The virus can only infect insects, so it is safe for mammals. As many types of cells can be transduced by AcMNPV, it is also a useful vector for gene delivery (Drugmand et al., 2012; Van Oers et al., 2015; Weissmann et al., 2016; Targovnik et al., 2021).

Over the past four decades, significant improvements to the original BEVS have been achieved. The establishment of bacmid system by incorporation of bacterial replicon into the viral genome has allowed quick and efficient generation of recombinant baculovirus (Luckow et al., 1993; Je et al., 2001a,b; Zhao et al., 2003). The production of multi-subunit protein complexes using a single baculovirus vector has been made possible by the development of MultiBac system (Berger et al., 2013). Alternatively, multiple foreign genes can be inserted at several loci within the baculovirus genome using multilocus baculovirus vectors (Kanai et al., 2013). These improvements have promoted the application potential of BEVS in the production of protein complexes in basic researches and virus like particles (VLPs) in the development of multivalent vaccines.

AcMNPV contains 155 predicted protein-coding genes (Chen T. et al., 2021). The functions of many of these genes are still unknown, and more than half of them are not required in the virus replication in cultured cells (Wang et al., 2007; Hitchman et al., 2010a; Chen T. et al., 2021; Yu et al., 2023). AcMNPV has two morphologically distinct virion types: budded virion (BV) and occlusion-derived virion (ODV). ODV is embedded in an alkaline-soluble protein matrix, which can maintain the stability of the virus particle in the environment and also be degradable in insect’s midgut, and therefore it is responsible for the primary infection or oral infection in insects. BVs produced from the primary infection will spread the infection to other cells and tissues, resulting in systemic infection of the host (Blissard and Theilmann, 2018). Commercially available BEVS usually lack the *polyhedrin* (*polh*) gene, as only BV is required for the infection of cultured cells. Besides polyhedrin, some other ODV-associated proteins are also nonessential for the production of BV and expression of recombinant proteins in cell culture (Rohrmann, 2019). In the development of baculoviruses as optimized vectors for gene delivery and expression, it has been found that removing some viral dispensable genes could not only generate smaller baculovirus genomes to accommodate larger exogenous gene fragments, but also improve the production of exogenous proteins. For example, deletion of *chitinase* (*chiA*) and *cathepsin* (*cath*) genes was found to be able to improve the stability of intracellular and secreted proteins and therefore increase the production of recombinant proteins (Kaba et al., 2004; Hitchman et al., 2010b). On this basis, a baculovirus vector that was deficient in *chiA*, *cath*, *p26*, *p10* and *p74* was constructed, and it was revealed that further deletion of *p26*, *p10* and *p74* had an additive beneficial effect on recombinant protein expression (Hitchman et al., 2010a). The bacmid with the deletion of all of the five nonessential genes is now commercially available (flashBACULTRA, Oxford Expression Technologies). In addition, knockout of *ODV-E26* has been shown to be able to effectively increase recombinant protein production in insect larvae (Pazmiño-Ibarra et al., 2019).

The function of many AcMNPV genes are not well understood, and the asymmetric distribution of essential and nonessential genes in the baculovirus genome provides ample opportunity for further improvement of the baculovirus vector by engineering the viral genome (Vijayachandran et al., 2013). Most of the existing gene-knockout studies focus on one or two single genes, such as *Ac18* (Wang et al., 2007), *Ac124* (Fang et al., 2019), *Ac68* and *lef3* (Nie et al., 2012), *Ac16* and *Ac17* (Nie and Theilmann, 2010), *Ac145* and *Ac150* (Lapointe et al., 2004). In terms of viral replication and BV production, *Ac18*, *Ac68*, *Ac124*, *Ac145* and *Ac150* are not important, while *lef3*, *Ac16* and *Ac17* are also nonessential but the gene deletion will delay or reduce viral replication. In a recent study, 42 AcMNPV genes whose functions were not fully understood were, respectively, knocked out, and 36 of them were found to be dispensable for BV production (Chen T. et al., 2021). A most recent study found that 51 AcMNPVs containing deletions of in total 62 nonessential genes had similar infectivity as the parental virus, and a potential minimal AcMNPV with a genome of 90 kb was proposed (Yu et al., 2023).

In this study, we deleted 14 DNA fragments from AcMNPV genome, each of them containing at least two contiguous genes that were known nonessential for viral replication in cell culture or functionally unknown. The effects of the fragment deletion on viral proliferation and foreign gene expression were examined. These data provided evidence for further improvement of BEVS by engineering the viral genome.

## 2. Materials and methods

### 2.1. Viruses and cell lines

*Spodoptera frugiperda* 9 (*Sf*9) cells (Invitrogen) were cultured in SFX insect medium (HyClone, GE healthcare life science) supplemented with 1% fetal bovine serum (HyClone). *Trichoplusia ni* (Tn) cells (High Five) were maintained in Serum-free insect cell culture medium at 27°C. Bacmid BAC10:KO_1629_ (Zhao et al., 2003) and Bac563-5 T (Zhang et al., 2021) were propagated in *E. coli* strain HS996. Plasmids pTriEx-GFP, pTriEx-OD-Fc and pTriEx-Fluc were stored in our laboratory (Zhang et al., 2021).

### 2.2. Construction of fragment-knockout bacmids and generation of recombinant baculoviruses

Baculovirus gene fragments were knocked out from the parental bacmid BAC10:KO_1629_ (named as bAc in this study) (Zhao et al., 2003) or Bac563-5 T (Zhang et al., 2021) using the Red/ET recombination system (the recombinase gene was introduced by pSC101 plasmid). RpsL-amp counter-selection cassette with homologous arms containing the target viral gene flanking sequences was amplified by PCR (Primer sequences are listed in Table 1). The target viral gene fragment was replaced with the amplified fragment that was transformed into HS996 competent cells, resulting in the corresponding fragment-knockout (KO) bacmid which was named as bAcΔX, where X represented the knockout genes in the DNA fragment.

**Table 1:** Primers employed in PCR.

Name	Primer sequence (5′-3′)
Ac1U	agttgaaggatcatatttagttgcgtttatgagataagattgaaagcacgtgtagatggcctggtgatgg
Ac5D	tcggcagttcttttgggcgtgatcgttgtcactacgggggtcatgtcatcgcttttaccaatgcttaatc
Ac1UT	gacattatccctcgattg
Ac11U	aaacaatctacatctatttcttcacaatccataacacacaacaggtccatcaatgatggcctggtgatgg
Ac13D	aacgcccaacaatggtcgcacatcgttaaatgggactcattcaaatgcaacacgttaccaatgcttaatc
Ac11UT	aagcatgatcgtgagtgg
Ac15U	tattcggtttgaagcaaaatgactattctctgctggcttgcactgctgtctacggatggcctggtgatgg
Ac16D	ccggcgactattttcataaactatgactcgcgaaccaaacgccgccgtcagcagttaccaatgcttaatc
Ac15UT	gcacactaacatgttgcc
Ac18U	gtattgttagaaaattgtgttgttttattagtataacgaaaaaatacatgacatgatggcctggtgatgg
Ac23D	atttatttcaattatacatgttttattttattctttctataatcatagggtacattaccaatgcttaatc
Ac18UT	actaaatgggttcctgcg
Ac29U	ttatttaaaaattgtctattccgtagttgagaaagttttgtcttgacttcataagatggcctggtgatgg
Ac33D	gacctggacacaaacgcgtacgaattgatggactttgagtaagatgcattcaccttaccaatgcttaatc
Ac29UT	gaaatcgacgaacgtgac
Ac44U	ttaagacgcaagcgcttcgagttttggcccgctcgctacctccgctgtacgactgatggcctggtgatgg
Ac49D	tcgtcgtaaaattagttgtatcaaagagcagctgcaattagaatcactgctaaattaccaatgcttaatc
Ac44UT	ctcaacaacatgcacgac
Ac55U	atttgttaaacaagcattcttatctcaataattggtccgacgtggtgacaattggatggcctggtgatgg
Ac61D	tgatatagttaatatggatcaatttgaacagttgattaacgtgtctctgctcaattaccaatgcttaatc
Ac55UT	aaacaaccagctacacgc
Ac63U	catgtacgataaatttatgatctatcttcacttgaatgggctgcacggagaagcgatggcctggtgatgg
Ac64D	ataacgtttttgaccaagattacgacagcggttattattccgattagtgttctattaccaatgcttaatc
Ac63UT	cacggttgtgttacagag
Ac68U	ccatattgctgttgtcgatatgtgggaatctatccgatggcaaatactgaatgggatggcctggtgatgg
Ac72D	taaaacattatgtacaataatatgtcttttattttacaatatttatgtatatgattaccaatgcttaatc
Ac68UT	cttgccttcttccatgtc
Ac84U	ctactcgtaaagcgagttgaattttgattacaaatattttgtttatgatagcaagatggcctggtgatgg
Ac87D	aactgtttacataacattctactttaatgtaataatattcttcaatttcttgggttaccaatgcttaatc
Ac84UT	cccttgatcatgcgttac
Ac96U	ttggctatcgtgtttgtacttttcgtgttaatttatttaataatttcgatcaaagatggcctggtgatgg
Ac97D	aaacattatggattacgaacaatattttattaaataaaaatataaactctattcttaccaatgcttaatc
Ac96UT	gttttgtcttgaggcacg
Ac114U	tgtcatcgtacaaactcgctttacgagtagaattctacgtgtaaaacacgattagatggcctggtgatgg
Ac122D	agcgacccatatattgtcgaatatagaacaccatgaaactgattatcctgctgtttaccaatgcttaatc
Ac122DT	gcatgattacagtcaacg
Ac129U	attgataagattatttttatctggctgttataaaaacgggatcatgaacacggagatggcctggtgatgg
Ac131D	cgttgatgtttgtgacgcttgacgctaaattggtcaaaatagagttggtgttgtttaccaatgcttaatc
Ac129UT	atacccgaccgttatctg
Ac148U	ttattatcgaggggccgttgttggtgtggggttttgcatagaaataacaatggggatggcctggtgatgg
Ac150D	tttatttttagttttggttagcggtacatccataatctgatataggaacacaatttaccaatgcttaatc
Ac148UT	attacagggtctggttcc
1st-UT	cacacaatatgaggacgc
1st-DT	agttatctacacgacggg

**Figure 1:**
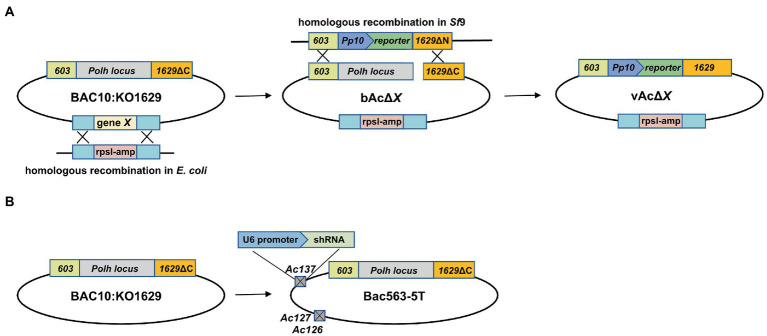
Schematic diagram of the construction of bacmids and generation of AcMNPV expressing a reporter gene. **(A)** Generation of fragment-knockout (KO) bacmid and AcMNPV expressing a reporter gene. DNA fragment containing predicted nonessential genes (gene X) was deleted from the AcMNPV bacmid BAC10:KO1629 by replacing the target fragment with a rpsl-Amp cassette *via* homologous recombination in *E. coli* strain HS996, and the KO bacmid was named as bAcΔX. The recombinant virus carrying the reporter gene expression cassette at the *polyhedrin* locus, vAcΔX, was generated in *Sf*9 cells by co-transfection with the linearized bAcΔX and pTriEx plasmid. The parental virus vAc generated from BAC10:KO1629 was used as a control in this study. **(B)** Schematic diagram of Bac563-5 T used in this study. Bac563-5 T was modified from BAC10:KO1629 by knocking out *ChiA/v-cath* (*Ac126-127*) and *p10* (*Ac137*) and inserting an shRNA expression cassette at the *Ac137* locus to inhibit the virus infection-induced apoptosis (Je et al., 2001a; Zhang et al., 2021).

To generate KO recombinant baculoviruses, a plasmid carrying a reporter gene was co-transfected with the linearized KO bacmid into *Sf*9 cells using FuGENE HD Transfection Reagent (Promega). Supernatants containing the recombinant baculoviruses were harvested at 5 days post transfection (dpt) by centrifugation at 300 × *g* for 5 min to remove cell debris. Viral titers were determined by 50% tissue culture infective doses (TCID_50_) assay after two passages. The approaches for the generation of KO Bacmids and recombinant viruses are illustrated in Figure 1.

### 2.3. Growth kinetic analysis

To compare the growth kinetics of different KO viruses (vAcΔX) with their parental virus (vAc), *Sf*9 cells were infected with the recombinant viruses expressing GFP at a multiplicity of infection (MOI) of 0.5. The supernatants of the infected cell cultures were harvested at 24, 48, 72, 96 and 120 h post infection (hpi). Titers of BV were determined by a TCID_50_ endpoint dilution assay in triplicates and the growth curves were made using GraphPad Prism software.

### 2.4. Protein expression and detection

*Sf*9 cells and High Five cells were infected with recombinant baculoviruses at an MOI of 3 and harvested for protein detection at 5 days post infection (dpi). Protein samples were separated by 10% sodium dodecyl sulfate-polyacrylamide gel electrophoresis (SDS-PAGE), followed by staining with Coomassie brilliant blue R-250. To calculate the expression level of target protein as a % of total cellular protein, protein bands were quantified by densitometry scanning using Image J software.

To monitor the expression of GFP in baculovirus infected cells, GFP fluorescence was observed by an inverted fluorescence microscope (Lecia DMI8) and the mean fluorescence intensity of the infected cells were analyzed by flow cytometry (NovoCyte 3000RYB).

Fc-tagged recombinant proteins were detected by Western blot using an HRP-conjugated goat anti-human antibody (CoWin Biotech). An enzyme-linked immunosorbent assay (ELISA) was used to quantify the secreted OD-Fc. Purified GP5-Fc (Wu et al., 2014), a recombinant protein containing the same Fc sequence, was used as a standard for the calibration of the protein concentration. Briefly, ELISA plates were individually coated with serial dilutions of GP5-Fc or cell culture medium containing OD-Fc, incubated at 4°C overnight, and then blocked with 5% skimmed milk in PBST at 37°C for 1 h. After three washes with PBST, HRP conjugated Goat Anti-Human IgG (CoWin Biotech) was added and incubated with the antigen for 1 h at 37°C. Unbound antibodies were washed away with PBST for three times. 3,3,5,5-tetramethylbenzidine (TMB) chromogenic substrate (Sangon Biotech) was added and incubated for 5 min at room temperature. The reaction was stopped by adding of H_2_SO_4_ and the absorbance was read at a wavelength of 450 nm using an Epoch Microplate Spectrophotometer (BioTek Epoch).

## 3. Results

### 3.1. Construction of AcMNPV fragment-knockout bacmids

AcMNPV encodes 155 ORFs, and more than half of the genes are non-essential for the production of BV. To construct smaller baculovirus genome for better expression of foreign genes and insertion of larger exogenous DNA fragments, we generated 14 fragment-knockout (KO) bacmids based on BAC10:KO1629 (Table 2). In each of them, a fragment containing at least two predicted nonessential genes (Je et al., 2001b; Chen T. et al., 2021; Yu et al., 2023) was replaced with the rpsl-amp counter-selection cassette by using the Red/ET recombination systems (Figure 1A). All the knockout fragments were confirmed by PCR (data not shown). In total, the 14 KO bacmids contained 62 knockout ORFs.

**Table 2:** Summary of the impact of the knockout of 14 DNA fragments on AcMNPV BV production and protein expression.

Fragment	ORFs	KO bacmid	KO Site	Length (bp)	Impact on BV production and protein expression
a	*Ac1-5*	bAcΔ1-5	501–2,936	2,436	Dispensable, ↑
b	*Ac11-13*	bAcΔ11-13	7,999–10,459	2,461	Essential
c	*Ac15-16*	bAcΔ15-16	11,462–13,619	2,158	Dispensable, ↑
d	*Ac18-23*	bAcΔ18-23	14,425–20,553	6,129	Essential
e	*Ac29-33*	bAcΔ29-33	24,096–28,281	4,186	Dispensable, ↑
f	*Ac44-49*	bAcΔ44-49	35,828–40,414	4,587	Dispensable, ↑
g	*Ac55-61*	bAcΔ55-61	46,400–49,116	2,717	Influential, ↓
h	*Ac63-64*	bAcΔ63-64	50,848–52,205	1,358	Dispensable, ↑
i	*Ac68-72*	bAcΔ68-72	58,929–61,980	3,052	Dispensable, ↑
j	*Ac84-87*	bAcΔ84-87	71,175–74,680	3,506	Dispensable, ↑
k	*Ac96-97*	bAcΔ96-97	84,415–85,003	589	Dispensable, ↑
l	*Ac114-122*	bAcΔ114-122	97,889–102,879	4,991	Essential
m	*Ac129-131*	bAcΔ129-131	109,911–111,661	1751	Dispensable, ↑
n	*Ac148-150*	bAcΔ148-150	129,059–130,708	1,650	Dispensable, ↓

↑ indicates that the reporter protein production was elevated. ↓ indicates that the reporter protein production was reduced.

### 3.2. Influences of fragment deletion on virus propagation in *Sf*9 cells

**Figure 2:**
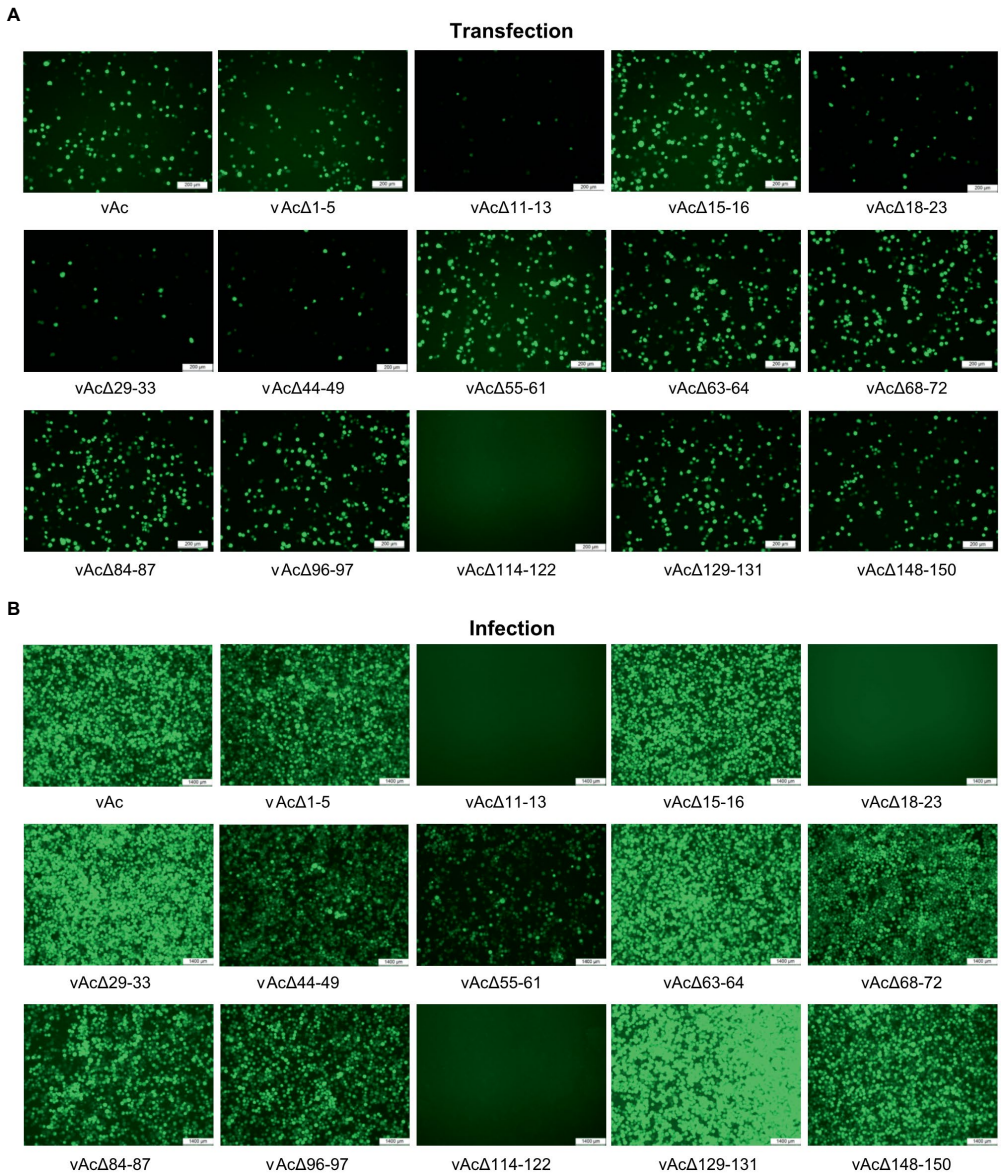
Characterization of KO AcMNPV expressing GFP by fluorescence microscopy. **(A)** The influence of the DNA fragment deletion on GFP expression after transfection. *Sf*9 cells were co-transfected with the KO bacmids and pTriEx-GFP, and the images were taken at 5 days post transfection (dpt). Bar, 200 μm. **(B)** The cells infected with the KO AcMNPV. The cell culture supernatants were harvested at 5 dpt and used to infect *Sf*9 cells, and the images were captured at 5 days post infection (dpi). Bar, 200 μm.

To define the effect of the fragment deletions on baculovirus replication, linearized KO bacmids and the parental bacmid were, respectively, co-transfected with pTriEx-GFP into *Sf*9 cells. The expression of GFP in the transfected *Sf*9 cells was visualized by using a fluorescence microscope at 5dpi (Figure 2A). GFP producing cells were observed in most of the wells, except that the cells transfected with bacmid bAcΔ114-122 had no fluorescence signal, suggesting that the deletion of *Ac114-122* eliminated the very late gene expression under the control of *p10* promoter. The subsequent infection results showed that deletions of *Ac11-13* and *Ac18-23*, in addition to *Ac114-122*, abolished the production of infectious BVs, indicating that essential genes were included in these three fragments. Infection of vAcΔ55-61 exhibited much less fluorescent cells than the parental virus vAc, hinting that BV production might be affected by the knockout of *Ac55-61*. The deletion of the rest 10 fragments did not damage the production of progeny viruses (Figure 2B).

### 3.3. Characterization of the replication kinetics of the KO viruses

**Figure 3:**
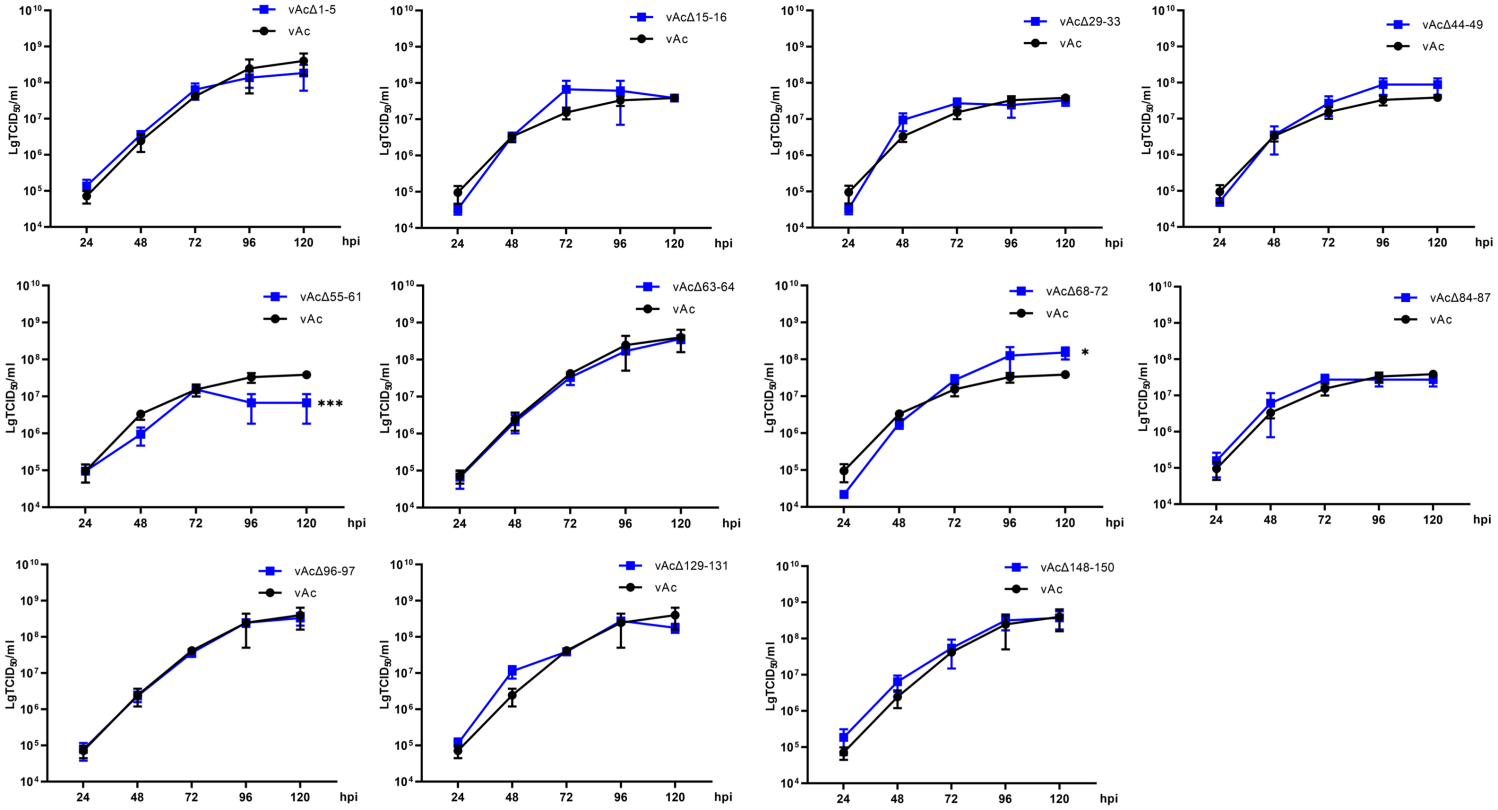
Growth kinetics curves. *Sf*9 Cells were infected by the virus at an MOI of 0.5. The cell culture supernatants from the infected cells were collected at intervals of 24 h, and BV titers were determined by an endpoint dilution assay in triplicate. *p < 0.05, ***p < 0.001 vs. vAc.

To characterize the replication kinetics of the KO viruses which can produce infectious BVs, supernatants from *Sf*9 cells infected with the recombinant viruses were titrated and used to infect cells at an MOI of 0.5, and then the titers of the viruses released in the cell culture supernatants were measured at intervals of 24 h until 120 hpi for the generation of growth kinetics curves (Figure 3). The results showed that vAcΔ1-5, vAcΔ15-16, vAcΔ29-33, vAcΔ44-49, vAcΔ63-64, vAcΔ84-87, vAcΔ96-97, vAcΔ129-131 and vAcΔ148-150 had similar growth curves as vAc. Compared with the parental virus, Further research is needed vAcΔ68-72 had higher titers of BVs and vAcΔ55-61 produced obviously lower levels of progeny BVs in late infection. Although the deletion of *Ac55-61* reduced the virus proliferation in late infection, the growth kinetics curves confirmed that the 43 genes included in the above 11 deleted fragments were nonessential for AcMNPV replication.

### 3.4. Production of green fluorescence protein

**Figure 4:**
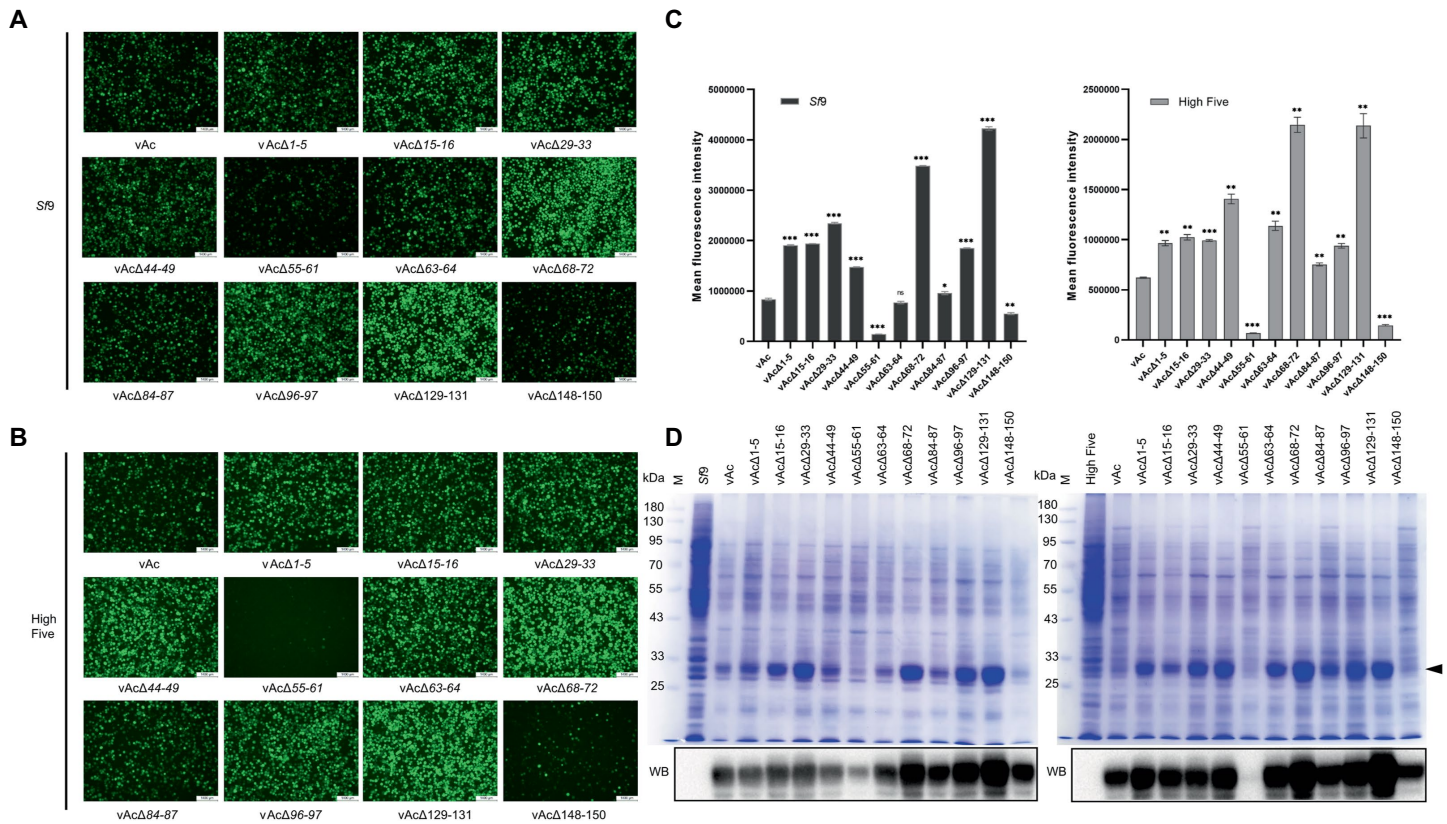
Production of fluorescence protein GFP in *Sf*9 and High Five cells. **(A)** Observation of reporter GFP expression in transfected *Sf*9 cells under fluorescence microscope. The cells were infected by the virus at an MOI of 3. **(B)** Observation of reporter GFP expression in High Five cells infected by the indicated viruses under fluorescence microscope. **(C)** Mean fluorescence intensity of GFP measured by flow cytometry. Data were collected from 3×10^4^ cells for each sample, and presented as the means ±SD of two replicates. ns: not significant, *p < 0.05, **p < 0.01, ***p < 0.001 vs. vAc. **(D)** Detection of the GFP protein levels by SDS-PAGE (upper panel) and Western blot (lower panel). The total proteins of the indicated cell lysates were separated by SDS-PAGE and visualized by coomassie brilliant blue R250 staining, and the GFP protein band is indicated by a black arrow. In Western blot analysis, the His-tagged recombinant GFP was probed by an anti-His monoclonal antibody.

To determine whether the KO bacmids can be used for improved expression of foreign gene, *Sf*9 cells and High Five cells were, respectively, infected by the recombinant viruses carrying GFP expression cassette driven by *p10* promoter at the *polh* locus at an MOI of 3. GFP fluorescence of the cells were monitored by an inverted fluorescence microscope (Figures 4A,B), and the mean fluorescence intensity was measured by flow cytometry at 5 dpi (Figure 4C). The results showed that the GFP fluorescence was significantly enhanced both in *Sf*9 and High Five cells infected by vAcΔ1-5, vAcΔ15-16, vAcΔ29-33, vAcΔ44-49, vAcΔ68-72, vAcΔ84-87, vAcΔ96-97 and vAcΔ129-131, compared to the control AcMNPV. Although the rise of GFP fluorescence was not significant in *Sf*9 cells infected with vAcΔ63-64, the fluorescence signal was significantly elevated in High Five cells. In cells infected with vAcΔ55-61 and vAcΔ148-150, the GFP fluorescence was obviously weakened, suggesting the severe suppression of GFP expression by the deletion of *Ac55-61* and *Ac148-150*.

SDS-PAGE and Western blot analyzes of the infected cell lysates showed that the GFP protein level was reduced more severely in vAcΔ55-61 infected cells than in vAcΔ148-150 infected cells. The results also confirmed that the protein production was elevated by most of the KO recombinant viruses (Figure 4D). The data demonstrated that the GFP production in BEVS could be obviously improved by deletion of some baculovirus fragments containing multiple non-essential genes.

### 3.5. Expression and secretion of glycoprotein OD-Fc

**Figure 5:**
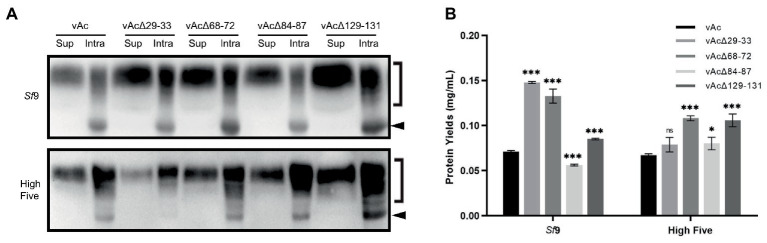
Expression of glycoprotein OD-Fc in *Sf*9 and High Five cells. **(A)** Detection of the intracellular and secreted OD-Fc by Western blot at 5 dpi. The unmodified target protein band is indicated by a black arrow, and the glycosylated form is indicated by a right square bracket. The OD-Fc protein was probed by HRP-conjugated anti-Human antibody in Western blot analysis. **(B)** Quantification of the level of OD-Fc secreted in the cell culture supernatant by ELISA at 5 dpi. Data were presented as the means ±SD of three replicates. ns: not significant, *p < 0.05, **p < 0.01, ***p < 0.001 vs. vAc.

To explore whether the knockout of non-essential genes could enhance extracellular production of secreted proteins, OD-Fc, a highly glycosylated recombinant protein containing the outer domain of HIV-1 gp120 (Chen et al., 2007; Zhang et al., 2021), was expressed using KO bacmids bAcΔ29-33, bAcΔ68-72 and bAcΔ129-131 that resulted in the best expression of GFP, and bAcΔ84-87 which only moderately improved the GFP production in High Five cells. The expression and secretion of OD-Fc was examined by Western blot at 5 dpi. The analysis detected both of the predicted molecular mass of unglycosylated OD-Fc (indicated by arrows in Figure 5A) and glycosylated OD-Fc (indicated by right square brackets in Figure 5A) in the infected cell lysates, and only the modified form of the protein appeared in the cell culture supernatant. The blotting results showed that the production and secretion of OD-Fc were generally elevated in *Sf*9 and High Five cells infected with the KO baculoviruses, except in the vAcΔ29-33 infected High Five cells. No obvious change in the molecular weight of the glycosylated form of protein was observed, suggesting that the glycosylation modification process was not affected by the viral gene deletions.

To better quantify the highly glycosylated and heterogeneous OD-Fc secreted in the cell culture media, ELISA analyzes were performed using purified GP5-Fc with the same Fc tag as a standard. Supernatant of the cell cultures infected with the recombinant baculoviruses was harvested for protein detection at 5 dpi. As shown in Figure 5B, the data confirmed that the production of secreted OD-Fc was significantly improved in both *Sf*9 and High Five cells infected with vAcΔ68-72 and vAcΔ129-131. The deletion of 29–33 resulted in two-fold increase of the yield of secreted OD-Fc in *Sf*9 cells but no significant improvement in High Five cells. In contrast, the production of secreted OD-Fc by vAcΔ84-87 was only significantly increased in High Five cells. These data demonstrate that knockout of some multiple genes which are nonessential for viral survival or infectivity are beneficial for the production of secreted glycoprotein in BEVS.

### 3.6. Protein production using combined fragment-knockout bacmids

In our previous report, we have developed a baculovirus vector Bac563-5 T carrying an shRNA expression cassette to inhibit virus infection-induced apoptosis and improve protein production in both *Sf*9 and High Five cells. In this vector, the nonessential *ChiA/v-cath* (*Ac126-127*) and *p10* (*Ac137*) genes have been deleted (Je et al., 2001a; Zhang et al., 2021). Based on Bac563-5 T, we investigated whether the dispensable fragment deletion could be combined to further enhance protein production in BEVS.

**Figure 6:**
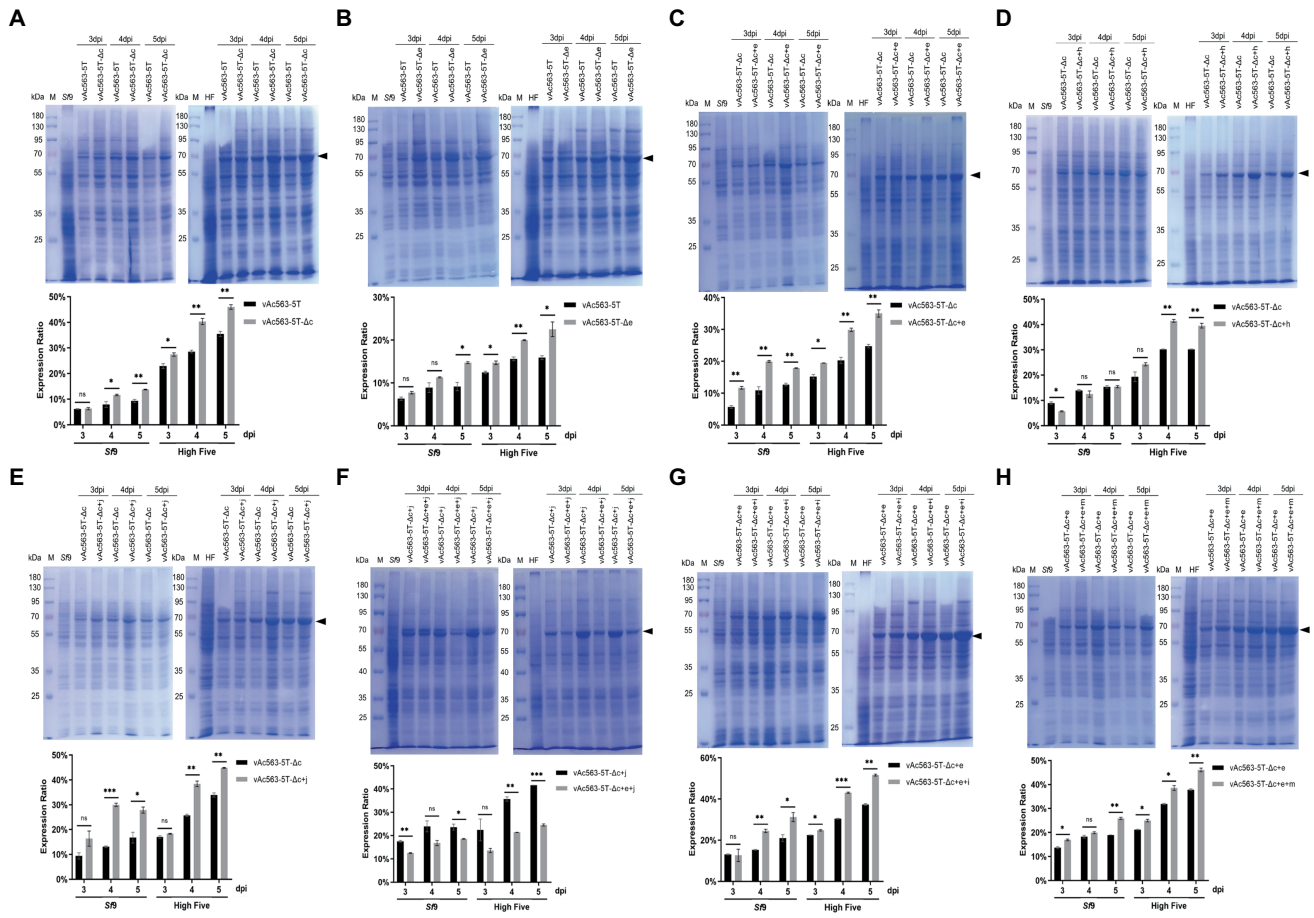
Expression of firefly luciferase (Fluc) using the indicated bacmid containing superimposed deletion of multiple nonessential fragments. **(A-H)** Impact of combination deletion of the indicated fragment on the Fluc expression. Cell lysates were harvested at 3, 4, and 5 dpi, separated by SDS-PAGE and visualized by coomassie brilliant blue R250 staining. The Fluc protein band is indicated by a black arrow. The protein bands were quantified by densitometry scanning using Image J software, and the expression levels of Fluc as a % of total cellular protein were calculated and the data were presented in the bar charts. *p < 0.05, **p < 0.01, ***p < 0.001.

By knocking out of *Ac15-16* and *Ac29-33, respectively,* from Bac563-5 T, we obtained Bac563-5 T-Δc and Bac563-5 T-Δe (Table 3). After generation of baculoviruses expressing firefly luciferase (Fluc) as a reporter protein, it revealed that neither the deletion of *Ac15-16* nor *Ac29-33* impacted the virus replication (Table 3), and both the fragment deletion benefited Fluc expression in *Sf*9 and High Five cells (Figures 6A,B).

**Table 3:** Summary of the combined deletions of nonessential fragments on Bac563-5 T.

KO bacmid	ORFs	Length (bp)	Virus titer (pfu/mL)
Bac563-5 T	*Ac126-127*, *Ac137*	2,213	9.25 × 10^7^ ± 3.62_a_
-Δc	*Ac126-127*, *Ac137*, *Ac15-16*	4,371	3.44 × 10^7^ ± 0.80_a_
-Δe	*Ac126-127*, *Ac137*, *Ac29-33*	6,399	6.74 × 10^7^ ± 1.12 _a_
-Δc + h	*Ac126-127*, *Ac137*, *Ac15-16*, *Ac63-64*	5,729	2.43 × 10^6^ ± 1.34_b_
-Δc + j	*Ac126-127*, *Ac137*, *Ac15-16*, *Ac84-87*	7,877	4.05 × 10^7^ ± 2.52_a_
-Δc + j + e	*Ac126-127*, Ac13*7, Ac15-16*, *Ac29-33*, *Ac84-87*	12,063	5.83 × 10^7^ ± 2.62_a_
-Δc + e	*Ac126-127*, *Ac137*, *Ac15-16*, *Ac29-33*	8,557	6.29 × 10^7^ ± 1.87_a_
-Δc + e + i	*Ac126-127*, *Ac137*, *Ac15-16*, *Ac29-33*, *Ac68-72*	11,609	3.88 × 10^7^_a_
-Δc + e + m	*Ac126-127*, *Ac137*, *Ac15-16*, *Ac29-33*, *Ac129-131*	10,308	4.89 × 10^7^ ± 1.74_a_

Virus titers were presented as the means ± SD of three replicates. Subscript letters indicate statistical significance using ANOVA followed by post hoc Student’s *t*-test (*p* < 0.05). ^a^indicates that the virus titer compared to Bac563-5T was not significantly different. ^b^indicates that the virus titer compared to Bac563-5T was significantly different.

Based on Bac563-5 T-Δc, the deletion of *Ac29-33*, *Ac63-64* and *Ac84-87* was, respectively, combined with the deletions of *Ac15-16*, *Ac126-127* and *Ac137*. The results showed that the virus titer decreased approximately one order of magnitude after the knockout of *Ac63-64* (Bac563-5 T-Δc + h), suggesting that combined deletion of *Ac63-64* with *Ac15-16*, *Ac126-127* and *Ac137* affected the virus replication ability, although the protein production was not reduced (Figure 6D). In contrast, the virus propagation ability was maintained (Table 3) and Fluc protein production was improved in the other two combinations (Bac563-5 T-Δc + e and Bac563-5 T-Δc + j) (Figures 6C,E). However, when both the deletions of *Ac29-33* and *Ac84-87* were combined on Bac563-5 T-Δc, the resulting Bac563-5 T-Δc + e + j had a lower protein yield than Bac563-5 T-Δc + j (Figure 6F).

As Bac563-5 T-Δc + e gave a higher average virus titer than Bac563-5 T-Δc + h, we further knocked out *Ac68-72* and *Ac129-131* from Bac563-5 T-Δc + e, generated Bac563-5 T-Δc + e + i and Bac563-5 T-Δc + e + m. Both of the bacmids maintained good virus propagation ability (Table 3). Notably, the yields of Fluc protein were close to 50% of total proteins in High Five cells and 30% in *Sf*9 cells (Figures 6G,H). Taken together, these data demonstrated that foreign protein production in BEVS could be noticeably increased by deletion of some large fragments containing multiple non-essential genes from AcMNPV genome.

## 4. Discussion

AcMNPV encodes about 150 open reading frames (ORFs) and the functions of many genes in viral replication and foreign protein expression are still unclear. Previous studies have suggested that more than half of the genes in AcMNPV and BmNPV baculovirus genomes are not required for the production of infectious BV when they are individually deleted (Ono et al., 2012; Chen T. et al., 2021; Yu et al., 2023). It has been proposed that the removal of redundant viral proteins may facilitate the virus to make better use of cell machineries and substrates to synthesize more target proteins, and it has been found that single and multiple deletions of some viral nonessential genes such as *polh*, *chiA*, *cath*, *p26*, *p10* and *p74* can increase recombinant protein yield without affecting the virus viability (Hitchman et al., 2010a). Whether deletion of large fragments containing other multiple nonessential genes could benefit the production of exogenous proteins remains to be investigated.

In this study, we systematically deleted 14 DNA fragments from AcMNPV genome to determine the effects on BEVS properties such as viral proliferation and foreign gene expression and secretion. The results revealed that deletion of *Ac1-5*, *Ac15-16*, *Ac29-33*, *Ac44-49*, *Ac55-61*, *Ac63-64*, *Ac68-72*, *Ac84-87*, *Ac96-97*, *Ac129-131* or *Ac148-150* was not lethal, demonstrating that the 43 genes included in the 11 fragments are nonessential for virus replication in cultured cells. AcMNPV mutants deleted for these fragments had normal replication ability in *Sf*9 cells, except that vAcΔ55-61 significantly produced less BVs than its parental virus. In AcMNPV and BmNPV, it has been shown that individual deletion of *Ac55* (*Bm44*), *Ac56* (*Bm45*), *Ac57* (*Bm46*), *Ac58/59* (*Bm47*) and *Ac60* (*Bm48*) does not affect the virus reproduction (Ono et al., 2012; Chen T. et al., 2021). For *Ac61*, its gene product FP25K is involved in nuclear trafficking of occlusion-associated proteins (Garretson et al., 2016). Studies have demonstrated that the gene knockout does not reduce BV production in BmNPV and AcMNPV (Ono et al., 2012; Garretson et al., 2016; Yu et al., 2023), but results in a ‘few polyhedra phenotype’ which are influenced by the host insect cells (Cheng et al., 2012). As virus mutants with single gene deletion in *Ac55-61* are normal for BV production, it is still unclear why vAcΔ55-61 has reduced BV productivity. It has been found that knockout of either ie-1 or ie-0 is not lethal for AcMNPV, but the mutant lacking the two genes lost the ability to produce BV (Stewart et al., 2005). It remains to be investigated whether there are functional compensation genes in *Ac55-61*.

For the other three AcMNPV mutants, the deletion of *Ac11-13*, *Ac18-23* and *Ac114-122* abolished the production of infectious BVs, indicating that these three fragments may contain essential genes for the virus replication. Among *Ac11-13*, *Ac11* is a highly conserved gene. Its homolog in BmNPV (*bm4*) is not associated with BV or ODV (Ono et al., 2012), and deletion of *Ac11* does not affect viral DNA replication, but it has been shown essential for infectious BV production and ODV envelopment in AcMNPV (Tao et al., 2015). The gene product can interact with components of the ESCRT-III complex and may be involved in the release of nucleocapsid at the nuclear membrane (Yue et al., 2018). *Ac12* has been identified dispensable for BV production (Chen T. et al., 2021), but a more recent study found that deletion of *Ac12* had a significant negative effect on the production of infectious viruses (Yu et al., 2023). *Ac13* is a conserved gene in all sequenced alphabaculoviruses and it encodes a late expression protein with a putative nuclear localization signal motif. Its gene product contains coiled-coil regions and has some structural similarity to some membrane proteins. Deletion of *Ac13* did not affect viral genome replication, nucleocapsid assembly or occlusion body (OB) formation but caused lower titers of BV due to lack of efficient nuclear egress from nucleus to cytoplasm (Chen X. et al., 2021). Its homolog in BmNPV (*bm5*) was found to be present in both the inner-and outer nuclear membranes (Nagamine et al., 2019). *Bm5* disruption resulted in lower titers of BV, fewer numbers of ODV and aberrant expression of various viral genes at the very late stage of infection (Kokusho et al., 2016). We tried to introduce *Ac11* back into vAcΔ11-13 and failed in the rescue of infectious virus particles (data not shown), which indicated that *Ac12* and *Ac13* cannot be simultaneously deleted from AcMNPV genome.

For *Ac18-23*, all the homolog genes have been demonstrated dispensable for BmNPV production (Ono et al., 2012), but a recent study shows that deletion of *Bm14* (homolog of *Ac23*) causes reduced BV and ODV production and also delayed death of infected larvae (Xu et al., 2019). *Ac23* encodes a fusion protein (F), which is involved in the formation of the BV envelope structure (Lung et al., 2003) and enhancing the infectivity of the budded virus (Wang et al., 2008). Reintroducing *Ac23* into vAcΔ18-23 in our study was not able to restore the virus infectivity (data not shown), suggesting that other genes in *Ac18-22* may also affect the production of infectious virus particles.

The design of *Ac114-122* deletion was based on the data that these genes were dispensable for BmNPV (Ono et al., 2012). However, our data here revealed that deletion of this fragment was lethal for AcMNPV. Consistent with our results, *Ac120* (Chen T. et al., 2021) and *Ac115* (Yu et al., 2023) were proven important for BV production in recent studies. It remains to be investigated whether re-introduction of *Ac115* and *Ac120* can restore the infectivity of vAcΔ114-122.

To test if the AcMNPV mutants deficient in multiple nonessential genes were appropriate vectors for the expression of exogenous proteins, we constructed recombinant baculoviruses for the intracellular expression of GFP and secretory production of glycoprotein OD-Fc. Among the 10 mutants with normal replication ability, most of them resulted in higher protein productivity, and only vAcΔ148-150 obviously gave a lower protein yield than the parental vector. Interestingly, some KO mutants exhibited cell type-dependent effects on protein production. Deletion of *Ac63-64* and *Ac84-87* resulted in more significant improvement of GFP production in High Five than in *Sf*9 cells. In contrast, lack of *Ac15-16*, *Ac29-33* and *Ac96-97* benefited the protein production more prominently in *Sf*9 cells than in High Five cells. The yield of secreted glycoprotein OD-Fc showed similar cell type-dependent tendency as the GFP production. Previous studies have discovered that *Sf*9 cells have a higher capacity for production of infectious virus particles, while High Five cells have higher susceptibility to baculovirus infection and a number of metabolic pathways can be hijacked to support mass production, therefore they exhibit higher protein productivity than *Sf*9 cells (Monteiro et al., 2014; Wilde et al., 2014). Our data here suggested that the effect of deletion of genes with different functions on the protein production in BEVS varied in different host cells. Consequently, the impacts of different cell lines should also be considered for developing the baculovirus expression vector system.

**Figure 7:**
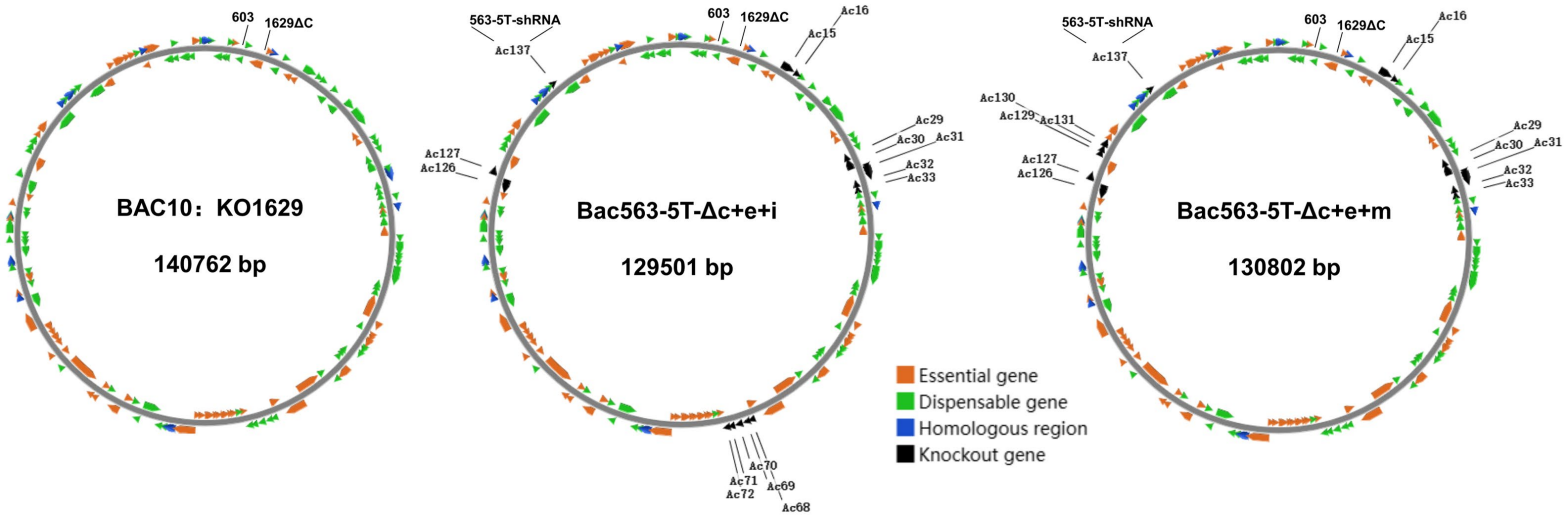
Schematic diagram of AcMNPV bacmid BAC10:KO1629 and the two shortened bacmids with improved capacity for recombinant protein production. BAC10:KO1629 (left) is 140,762 bp, containing 154 AcMNPV ORFs and a F replicon (Kan^r^) fragment inserted between ORF603 and truncated ORF1629. Compared with BAC10:KO1629, the shortened Bac563-5 T-Δc + e + i containing deletions of *Ac15-16*, *Ac29-33*, *Ac68-72*, *Ac126-127* and *Ac137* (middle) and Bac563-5 T-Δc + e + m containing deletions of *Ac15-16*, *Ac29-33*, A*c126-127*, *Ac129-131* and *Ac137* (right) exhibit similar replication ability and better protein productivity.

Based on our previously constructed Bac563-5 T with the knockout of *ChiA/v-cath* (*Ac126-127*) and *p10* (*Ac137*) (Je et al., 2001a; Zhang et al., 2021), we tried to delete more dispensable fragments from this Bacmid. Despite the fact that individual deletion of these nonessential fragments increased protein expression, simultaneous knockout of them may have an adverse impact on protein expression, and distinct combinations of deletions may have diverse effects on protein expression. In the end, we successfully generated three bacmid vectors each containing three more dispensable fragments removed. Compared with BAC10:KO_1629_ (Zhao et al., 2003), all these three bacmids are more than 10 kb shorter in length but the viruses have comparable replication capabilities in *Sf*9. Among them, Bac563-5 T-Δc + e + j had reduced protein production than Bac563-5 T-Δc + e after the deletion of *Ac84-87*, but Bac563-5 T-Δc + e + i (with combined deletion of *Ac15-16*, *Ac29-33*, *Ac68-72*, *Ac126-127* and *Ac137*) and Bac563-5 T-Δc + e + m (with combined deletion of *Ac15-16*, *Ac29-33*, *Ac126-127*, *Ac137* and *Ac129-131*) showed improved protein expression performance (maps illustrating the deletions in the genome are shown in Figure 7). In *Sf*9 cells, the amount of expressed Fluc occupied around 30% of the total intracellular proteins at 5dpi, while in High five cells, the ratio was close to 50% of the total proteins. More efforts are needed to investigate whether more dispensable fragments can be removed from the AcMNPV genome to maintain or further improve the protein productivity in BEVS.

Nowadays, the re-design and *de novo* synthesis of viral genome techniques have become mature. Since the first synthetic baculovirus was rescued in 2017, several re-designed AcMNPV have been constructed (Shang et al., 2017, 2021; Guo et al., 2022). In a recent report, a synthetic AcMNPV with 17 kb deletion in the C1 Region of the viral genome was generated, but the titer of infectious virus particles produced in *Sf*9 cells dropped two orders of magnitude (Guo et al., 2022). Synthetic virology provides a powerful platform for the understanding of baculovirus replication and the application of the virus as gene delivery and protein expression vectors. Compared to the traditional top-down strategy used in this study, it could be easier and quicker to generate a new viral genome containing multiple deletions or modifications using the bottom-up synthetic approach. However, the design a minimal baculovirus with comparable or better replication ability and protein productivity still requires more basic research on baculovirus biology for better understanding of the roles of viral proteins in the virus replication.

## 5. Conclusion

In this study, we constructed 14 fragment-knockout mutants of AcMNPV, each having at least two genes deleted, and identified 11 of the fragments containing 43 genes could be individually deleted without abolishing BV production. Using the AcMNPV mutants deficient in multiple nonessential genes as vector for exogenous protein expression, the results demonstrated that nine of the fragment-deletions benefited protein production. By combining the deletion of fragments dispensable for virus replication and recombinant protein expression, we obtained two AcMNPV vectors which were shortened more than 10 kb and simultaneously displayed improved capacity for recombinant protein production. This report could serve as a foundation for further improvement of the BEVS as a platform for protein production in biopharmaceutical industry and basic researches.

## Data availability statement

The raw data supporting the conclusions of this article will be made available by the authors, without undue reservation.

## Author contributions

HC, XX, and XZ conceived the study and designed the experiments. XZ, AH, YZ, HT, and KZ performed the experiments. XZ and ZZ analyzed the data. HC, XZ, and AH wrote the manuscript. All authors have read and approved the final manuscript.

## Funding

This work was sponsored by Yangling science and technology project (2016SF-06), the Natural Science Basic Research Plan in Shaanxi Province of China (2021JM-098) and in part by Shaanxi Bacmid Biotechnology Co., Ltd. (K4050422140).

## Conflict of interest

XX is the founder and a shareholder of this company. Patents covering the AcMNPV vector described in this manuscript are as follows: patent ZL 2019 10650551.5 is owned by Northwest A&F University, and patents pending 2021103927781, 2021100308313, 2021103924181, 2021107427173, and 202110408273X are owned by Shaanxi Bacmid Biotechnology Co., Ltd.

The remaining authors declare that the research was conducted in the absence of any commercial or financial relationships that could be construed as a potential conflict of interest.

## Publisher’s note

All claims expressed in this article are solely those of the authors and do not necessarily represent those of their affiliated organizations, or those of the publisher, the editors and the reviewers. Any product that may be evaluated in this article, or claim that may be made by its manufacturer, is not guaranteed or endorsed by the publisher.

## References

[ref1] Berger I. Garzoni F. Chaillet M. Haffke M. Gupta K. Aubert A. (2013). The Multibac protein complex production platform at the Embl. J. Vis. Exp. 77:E50159. doi: 10.3791/50159, PMID: 23892976 PMC3747712

[ref2] Blissard G. W. Theilmann D. A. (2018). Baculovirus entry and egress from insect cells. Annu. Rev. Virol. 5, 113–139. doi: 10.1146/Annurev-Virology-092917-043356, PMID: 30004832

[ref3] Chen T. Duan X. Hu H. Shang Y. Hu Y. Deng F.. (2021). Systematic analysis of 42 *Autographa californica multiple nucleopolyhedrovirus* genes identifies An additional six genes involved in the production of infectious budded virus. Virol. Sin. 36, 762–773. doi: 10.1007/S12250-021-00355-1, PMID: 33683665 PMC8379328

[ref4] Chen H. Xu X. Jones I. M. (2007). Immunogenicity of the outer domain of a Hiv-1 clade C Gp120. Retrovirology 4:33. doi: 10.1186/1742-4690-4-33, PMID: 17509143 PMC1891314

[ref5] Chen X. Yang X. Lei C. Qin F. Sun X. Hu J. (2021). *Autographa californica multiple nucleopolyhedrovirus* Orf13 is required for efficient nuclear egress of nucleocapsids. Virol. Sin. 36, 968–980. doi: 10.1007/S12250-021-00353-3, PMID: 33721216 PMC8558143

[ref6] Cheng X. Kumar S. Arif B. Krell P. Zhang C. Cheng X. (2012). Cell-dependent polyhedra production and Virion occlusion of Acmnpv Fp25k mutants in vitro and in vivo. J. Gen. Virol. 94, 177–186. doi: 10.1099/Vir.0.045591-0, PMID: 22993192

[ref7] Drugmand J.-C. Schneider Y.-J. Agathos S. N. (2012). Insect cells as factories for biomanufacturing. Biotechnol. Adv. 30, 1140–1157. doi: 10.1016/J.Biotechadv.2011.09.014, PMID: 21983546

[ref8] Fang Z. Que Y. Li J. Zhang Z. (2019). The deletion of the Acmnpv Ac124 gene resulted in a decrease in Chitinase transcription. Virus Res. 263, 151–158. doi: 10.1016/J.Virusres.2019.01.017, PMID: 30711578

[ref9] Garretson T. A. Mccoy J. C. Cheng X.-W. (2016). Baculovirus Fp25k localization: role of the coiled-coil domain. J. Virol. 90, 9582–9597. doi: 10.1128/Jvi.01241-16, PMID: 27512078 PMC5068512

[ref10] Guo Y. Hu H. Xiao H. Deng F. Li J. Wang M.. (2022). Successful rescue of synthetic Acmnpv with a ~17 kb deletion in the C1 region of the genome. Viruses 14:2780. doi: 10.3390/V14122780, PMID: 36560785 PMC9782167

[ref11] Hitchman R. B. Possee R. D. Crombie A. T. Chambers A. Ho K. Siaterli E.. (2010a). Genetic modification of a baculovirus vector for increased expression in insect cells. Cell Biol. Toxicol. 26, 57–68. doi: 10.1007/S10565-009-9133-Y, PMID: 19655260

[ref12] Hitchman R. B. Possee R. D. Siaterli E. Richards K. S. Clayton A. J. Bird L. E.. (2010b). Improved expression of secreted and membrane-targeted proteins in insect cells. Biotechnol. Appl. Biochem. 56, 85–93. doi: 10.1042/Ba20090130, PMID: 20441568

[ref13] Je Y. H. Chang J. H. Choi J. Y. Roh J. Y. Jin B. R. O'reilly D. R.. (2001a). A defective viral genome maintained in Escherichia Coli for the generation of baculovirus expression vectors. Biotechnol. Lett. 23, 575–582. doi: 10.1023/A:1010301404445

[ref14] Je Y. H. Chang J. H. Roh J. Y. Jin B. R. (2001b). Generation of Baculovirus expression vector using detective Autographa California nuclear Polyhedrosis virus genome maintained in *Escherichia coli* for Occ+ virus production. Int. J. Indust. Entomol 2, 155–160.

[ref15] Kaba S. A. Salcedo A. M. Wafula P. O. Vlak J. M. Van Oers M. M. (2004). Development of a Chitinase and V-Cathepsin negative Bacmid for improved integrity of secreted recombinant proteins. J. Virol. Methods 122, 113–118. doi: 10.1016/J.Jviromet.2004.07.006, PMID: 15488628

[ref16] Kanai Y. Athmaram T. Stewart M. Roy P. (2013). Multiple large foreign protein expression by a single recombinant baculovirus: a system for production of multivalent vaccines. Protein Expr. Purif. 91, 77–84. doi: 10.1016/J.Pep.2013.07.005, PMID: 23872366

[ref17] Kokusho R. Koh Y. Fujimoto M. Shimada T. Katsuma S. (2016). *Bombyx mori* Nucleopolyhedrovirus Bm5 protein regulates progeny virus production and viral gene expression. Virology 498, 240–249. doi: 10.1016/J.Virol.2016.08.032, PMID: 27614700

[ref18] Lapointe R. Popham H. J. Straschil U. Goulding D. O'reilly D. R. Olszewski J. A. (2004). Characterization of two Autographa californica nucleopolyhedrovirus proteins, Ac145 and Ac150, which affect Oral infectivity in a host-dependent manner. J. Virol. 78, 6439–6448. doi: 10.1128/Jvi.78.12.6439-6448.2004, PMID: 15163737 PMC416519

[ref19] Luckow V. A. Lee S. C. Barry G. F. Olins P. O. (1993). Efficient generation of infectious recombinant baculoviruses by site-specific transposon-mediated insertion of foreign genes into a Baculovirus genome propagated in *Escherichia coli*. J. Virol. 67, 4566–4579. doi: 10.1128/Jvi.67.8.4566-4579.1993, PMID: 8392598 PMC237841

[ref20] Lung O. Y. Cruz-Alvarez M. Blissard G. W. (2003). Ac23, An envelope fusion protein homolog in the baculovirus *Autographa californica multicapsid nucleopolyhedrovirus*, is a viral pathogenicity factor. J. Virol. 77, 328–339. doi: 10.1128/Jvi.77.1.328-339.2003, PMID: 12477838 PMC140606

[ref21] Monteiro F. Bernal V. Saelens X. Lozano A. B. Bernal C. Sevilla A.. (2014). Metabolic profiling of insect cell lines: unveiling cell line determinants behind System's productivity. Biotechnol. Bioeng. 111, 816–828. doi: 10.1002/Bit.25142, PMID: 24258249

[ref22] Nagamine T. Inaba T. Sako Y. (2019). A nuclear envelop-associated Baculovirus protein promotes Intranuclear lipid accumulation during infection. Virology 532, 108–117. doi: 10.1016/J.Virol.2019.04.006, PMID: 31055062

[ref23] Nie Y. Fang M. Erlandson M. A. Theilmann D. A. (2012). Analysis of the *Autographa californica multiple nucleopolyhedrovirus* overlapping gene pair Lef3 and Ac68 reveals that Ac68 is a per Os infectivity factor and that Lef3 is critical, but not essential, for virus replication. J. Virol. 86, 3985–3994. doi: 10.1128/Jvi.06849-11, PMID: 22278232 PMC3302525

[ref24] Nie Y. Theilmann D. A. (2010). Deletion of Acmnpv Ac16 and Ac17 results in delayed viral gene expression in budded virus infected cells but not transfected cells. Virology 404, 168–179. doi: 10.1016/J.Virol.2010.03.031, PMID: 20627351

[ref25] Ono C. Kamagata T. Taka H. Sahara K. Asano S.-I. Bando H. (2012). Phenotypic grouping of 141 Bmnpvs lacking viral gene sequences. Virus Res. 165, 197–206. doi: 10.1016/J.Virusres.2012.02.016, PMID: 22421381

[ref26] Pazmiño-Ibarra V. Mengual-Martí A. Targovnik A. M. Herrero S. (2019). Improvement of Baculovirus as protein expression vector and as biopesticide by Crispr/Cas9 editing. Biotechnol. Bioeng. 116, 2823–2833. doi: 10.1002/Bit.27139, PMID: 31403180

[ref27] Rohrmann G. F.. (2019). Baculovirus Molecular Biology. 4th, Bethesda, MD: National Center For Biotechnology Information (Us). 31294936

[ref28] Shang Y. Hu H. Wang X. Wang H. Deng F. Wang M.. (2021). Construction and characterization of a novel Bacmid Acbac-Syn based on a synthesized Baculovirus genome. Virol. Sin. 36, 1566–1574. doi: 10.1007/S12250-021-00449-W, PMID: 34569015 PMC8692530

[ref29] Shang Y. Wang M. Xiao G. Wang X. Hou D. Pan K.. (2017). Construction and rescue of a functional synthetic Baculovirus. ACS Synth. Biol. 6, 1393–1402. doi: 10.1021/Acssynbio.7b00028, PMID: 28383905

[ref30] Smith G. E. Summers M. D. Fraser M. (1983). Production of human Beta interferon in insect cells infected with a Baculovirus expression vector. Mol. Cell. Biol. 3, 2156–2165. doi: 10.1128/Mcb.3.12.2156-2165.1983, PMID: 6318086 PMC370086

[ref31] Stewart T. M. Huijskens I. Willis L. G. Theilmann D. A. (2005). The *Autographa californica multiple nucleopolyhedrovirus* Ie0-Ie1 gene complex is essential for wild-type virus replication, but either Ie0 or Ie1 can support virus growth. J. Virol. 79, 4619–4629. doi: 10.1128/Jvi.79.8.4619-4629.2005, PMID: 15795248 PMC1069578

[ref32] Tao X. Y. Choi J. Y. Kim W. J. An S. B. Liu Q. Kim S. E.. (2015). *Autographa californica multiple nucleopolyhedrovirus* Orf11 is essential for budded-virus production and occlusion-derived-virus envelopment. J. Virol. 89, 373–383. doi: 10.1128/Jvi.01742-14, PMID: 25320313 PMC4301119

[ref33] Targovnik A. M. Simonin J. A. Mc Callum G. J. Smith I. Cuccovia Warlet F. U. Nugnes M. V.. (2021). Solutions against emerging infectious and noninfectious human diseases through the application of Baculovirus technologies. Appl. Microbiol. Biotechnol. 105, 8195–8226. doi: 10.1007/S00253-021-11615-1, PMID: 34618205 PMC8495437

[ref34] Van Oers M. M. Pijlman G. P. Vlak J. M. (2015). Thirty years of Baculovirus–insect cell protein expression: from dark horse to mainstream technology. J. Gen. Virol. 96, 6–23. doi: 10.1099/Vir.0.067108-0 25246703

[ref35] Vijayachandran L. S. Thimiri Govinda Raj D. B. Edelweiss E. Gupta K. Maier J. Gordeliy V.. (2013). Gene gymnastics: synthetic biology for Baculovirus expression vector system engineering. Bioengineered 4, 279–287. doi: 10.4161/Bioe.22966, PMID: 23328086 PMC3813527

[ref36] Wang M. Tan Y. Yin F. Deng F. Vlak J. M. Hu Z.. (2008). The F-like protein Ac23 enhances the infectivity of the budded virus of Gp64-null *Autographa californica multinucleocapsid nucleopolyhedrovirus* Pseudotyped with baculovirus envelope fusion protein F. J. Virol. 82, 9800–9804. doi: 10.1128/Jvi.00759-08, PMID: 18653446 PMC2546973

[ref37] Wang Y. Wu W. Li Z. Yuan M. Feng G. Yu Q.. (2007). Ac18 is not essential for the propagation of *Autographa californica multiple nucleopolyhedrovirus*. Virology 367, 71–81. doi: 10.1016/J.Virol.2007.05.017 17573091

[ref38] Weissmann F. Petzold G. VanderLinden R. Huis in 't Veld P. J. Brown N. G. Lampert F.. (2016). Bigbac enables rapid gene assembly for the expression of large multisubunit protein complexes. Proc. Natl. Acad. Sci. U. S. A. 113, E2564–E2569. doi: 10.1073/Pnas.1604935113, PMID: 27114506 PMC4868461

[ref39] Wilde M. Klausberger M. Palmberger D. Ernst W. Grabherr R. (2014). Tnao38, high five and Sf9--evaluation of host-virus interactions in three different insect cell lines: baculovirus production and recombinant protein expression. Biotechnol. Lett. 36, 743–749. doi: 10.1007/S10529-013-1429-6, PMID: 24375231 PMC3955137

[ref40] Wu F. Peng K. Tian J. Xu X. Zhou E. Chen H. (2014). Immune response to fc tagged Gp5 glycoproteins of porcine reproductive and respiratory syndrome virus. Viral Immunol. 27, 343–349. doi: 10.1089/Vim.2014.0041, PMID: 25014350

[ref41] Xu W. Fan Y. Wang H. Feng M. Wu X. (2019). *Bombyx mori* nucleopolyhedrovirus F-like protein Bm14 affects the morphogenesis and production of occlusion bodies and the embedding of Odvs. Virology 526, 61–71. doi: 10.1016/J.Virol.2018.10.008, PMID: 30342303

[ref42] Yu Y. Zhang T. Lu D. Wang J. Xu Z. Zhang Y.. (2023). Genome-wide nonessential gene identification of *Autographa californica multiple nucleopolyhedrovirus*. Gene 863:147239. doi: 10.1016/J.Gene.2023.147239 36736504

[ref43] Yue Q. Yu Q. Yang Q. Xu Y. Guo Y. Blissard G. W.. (2018). Distinct roles of cellular Escrt-I and Escrt-iii proteins in efficient entry and egress of budded Virions of *Autographa californica multiple nucleopolyhedrovirus*. J. Virol. 92, E01636–E01617. doi: 10.1128/Jvi.01636-17 29046462 PMC5730794

[ref44] Zhang X. Zhao K. Lan L. Shi N. Nan H. Shi Y.. (2021). Improvement of protein production by engineering a novel antiapoptotic baculovirus vector to suppress the expression of sf-Caspase-1 and Tn-Caspase-1. Biotechnol. Bioeng. 118, 2977–2989. doi: 10.1002/Bit.27807, PMID: 33990946

[ref45] Zhao Y. Chapman D. A. Jones I. M. (2003). Improving baculovirus recombination. Nucleic Acids Res. 31, 6e–66e. doi: 10.1093/Nar/Gng006, PMID: 12527795 PMC140531

